# Suppression of the FA pathway combined with CHK1 inhibitor hypersensitize lung cancer cells to gemcitabine

**DOI:** 10.1038/s41598-017-15172-4

**Published:** 2017-11-08

**Authors:** Chun-Hua Dai, Yi Wang, Ping Chen, Qian Jiang, Ting Lan, Mei-Yu Li, Jin-Yu Su, Yan Wu, Jian Li

**Affiliations:** 1 Department of Radiation Oncology, Affiliated Hospital of Jiangsu University, Zhenjiang, China; 2 Center of Experimental Medicine, Affiliated Hospital of Jiangsu University, Zhenjiang, China; 3 Department of Pulmonary Medicine, Affitialed Hospital of Jiangsu University, Zhenjiang, China; 4 Institute of Medical Science, Jiangsu University, Zhenjiang, Jiangsu China

## Abstract

The combination of platinum and gemcitabine is one of the standard regimens in the treatment of advanced lung squamous carcinoma (LSC). Resistance to gemcitabine is main barrier to the successful treatment of LSC. In this study, we showed that suppression of the Fanconi anemia (FA) pathway increased the sensitivity of two LSC cell lines SK-MES-1 and KLN205 to gemcitabine. Moreover, we found that the CHK1 pathway and the FA pathway are functionally compensatory in the repair of DNA damage in the LSC cell lines. Inactivation of one of the two pathways led to DNA damage, triggering compensatory activation of other pathway. Furthermore, we demonstrated that FANCD2 depletion combined with CHK1 inhibitor MK-8776 significantly potentiated the cytotoxicity of gemcitabine to the two LSC cell lines, compared to individual FANCD2 depletion or MK-8776 treatment. The enhanced effect of gemcitabine-chemosensitization was accompanied by loss of DNA repair function and accumulation of DNA single strand breaks and double strand breaks, in parallel with obvious increase of caspase-3 dependent apoptosis. Our results indicate that the enhancement effect of FANCD2 depletion combined with CHK1 inhibitor in sensitizing the LCS cells to gemcitabine supports the FA pathway and CHK1 as two therapeutic targets for improvement of anti-tumor regimens in treatment of LSC.

## Introduction

Lung cancer is the top cause of cancer-related death^1^. Non-small cell lung cancer (NSCLC) accounts for about 85% of all lung cancer and more than 60% of NSCLC patients are diagnosed in locally advanced and advanced stage^2,3^. Although the discovery of targeted drugs has led to improvements in NSCLC treatment for patients with sensitizing EGFR mutation positive or ALK rearrangement positive, targeted drugs are only efficacious in a subset of NSCLC patients and their long-term use ultimately result in drug resistance and disease recurrent^4,5^. Thus chemotherapy still play important role in the management of advanced NSCLC. The combination of platinum and gemcitabine has been used in clinic as one of the standard regimens for lung squamous carcinoma (LSC)^6^. A number of clinical trials have attempted to improve gemcitabine-containing regimen chemotherapy^7–9^, but the inherent or acquired resistance to gemcitabine is main barrier to the successful treatment of LSC. It is important to probe the mechanism of gemcitabine resistance and the approach of overcoming resistance.

Gemcitabine inhibits ribonucleotide reductase depleting the cellular pool of deoxyribonucleotides and stalling replication fork progression^10^. In addition, gemcitabine can be incorporated into the growing DNA strand, and induces chain termination after the addition of the next nucleotide^11^. These perturbations of DNA metabolism prevent complete replication and trigger the DNA damage response (DDR) pathways^12^. Replicative block from gemcitabine treatment activates the ATR/CHK1 pathway. CHK1 is a central mediator of the cellular response to DNA damage^13^. Activation of CHK1 through phosphorylation of its ser-317 and ser-345 by ATR results in inhibition of Cdc25 phosphatases and cell cycle arrest at the S and/or G2/M phases^14^. Also CHK1 contributes to DDR by regulating the RAD51-mediated homologous recombination repair (HRR)^15^. Inhibition of CHK1 with either siRNA or chemical inhibitors prevents drugs-induced Cdc25 degradation, leading to abrogation of the S and/or G2/M phase checkpoints and premature mitosis^16–18^, and potentiates the cytotoxicity of genotoxic agents *in vitro* and *in vivo* ^19–21^.

It has been recognized that DDR checkpoint and especially CHK1 activity may be critical for keeping normal replication of cancer cells with specific defects in the DNA repair or DDR pathways. For instance, the Fanconi anemia (FA) pathway is responsible for repairing DNA crosslinks and double strand breaks (DSBs) and maintaining chromosomal stability^22^. FA deficient cells were found to be sensitive to CHK1 inhibition by siRNA compared to FA proficient cells^23^. We hypothesized that co-inhibition of CHK1 and the FA pathway might further sensitize LSC cells to gemcitabine compared to the suppression of CHK1 or the FA pathway alone.

In this study, we showed that silencing of the FA genes led to activation of CHK1 and vice versa, as well as the sensitization to CHK1 inhibitor MK-8776 (previously known as SCH900776) and gemcitabine in LSC cell lines. Furthermore, combination of MK-8776 and the FA pathway suppression by siRNA produced significant gemcitabine-sensitization as compared with CHK1 inhibitor treatment and the FA pathway suppression alone, which was accompanied by markedly elevated phosphorylations of CHK1 (S345), CHK2 (T68), and H2AX (S139), and obvious inhibition of the RAD51-mediated repair of gemcitabine-induced DSBs, demonstrating a enhancement effect between CHK1 inhibitor and suppression of the FA pathway in sensitizing LSC cells to gemcitabine.

## Materials and Methods

### Cell culture, and chemicals and reagents

NSCLC cell lines A549, HCC4006 and Calu-1 (three human lung adenocarcinoma cell lines), and SK-MES-1 and KLN205 (two human lung squamous carcinoma cell lines) were obtained from the Shanghai Institute for Biological Science (China). All cell lines were purchased between 2012 and 2015 and authenticated based on growth rate, morphology, and viability and were frequently confirmed to be mycoplasma free. The cells were cultured in RPMT 1640 media supplemented with 10% heat-inactivated fetal calf serum (FBS), L-glutamine and 100 U/ml penicillin, and 100 μg/ml streptomycin sulfate. The cells were maintained in a humidified incubator in 5% CO_2_ at 37 °C.

Antibodies to various antigens were as follows: CHK1, PCHK1 (S296), PCHK1 (S317), PCHK1 (S345), CHK2, PCHK2 (T68), PCHK2 (S516), Cdc25A, PCdc25C (S216), H2AX, γH2AX (S139), PH3 (S10histone), ATM, PATM (S1981), DNA-PKCs, PDNA-PKCs (S2056), and GAPDH were from Cell Signaling. FANCL, FANCD2, BRCA2, PARP1, RAD51, caspase-3, cleaved caspanse-3, PARP and cleaved PARP from Santa Cruz. Gemcitabine was from Hanson Pharmaceutic (China), Cisplatin from Yangtze River Pharmaceutic (China), MK-8776 from Selleck Chemicals, CHK2 inhibitor II from Abcam. Gemcitabine was dissolved in PBS. Cisplatin, MK-8776 and CHK2 inhibitor II were dissolved in DMSO and used at the specified concentrations.

### Cell viability and colony formation

Cell viability was determined by the cell counting-kit (CCK-8) assay after siRNA transfections or drug treatments according to manufacture’s instructions as previously described^24^. For detection of clonogenic ability of the cells with drug treatment, after siRNA transfections, a density of 1000 cells per cell seed onto a 6-well culture plate and the indicated drugs at various doses or vehicle in DMEM containing 10% FBS was added to the cultures at 3 day after seeding. The cultures were continuously maintained for another two weeks and subject to the colony formation assay. Colonies produced by each cell-group were counted and measured using Image Software^25^.

### Transfections with siRNAs

The following siRNA target sequences were used:

FANCL-1 (5′-CTTGCTGTGTGACTGTCAC-3′), FANCL-2 (5′-CCAGGAAGCAACCACTTTC-3′), FANCD2-1 (5′-GCTCTTTCAACGTAAATTA-3′), FANCD2-2 (5′-GGTTGGCTTAATAAATTCA-3′),

BRCA2-1 (5′-GTTTAGAAAGCCAAGCTACTA-3′); BRCA2-2 (5′-GACUCUAGGUCAAGAUUUAAG-3′).

The indicated siRNAs were synthesized by Guangzhou Riboio Co., Ltd (China). siRNAs were transfected using Lipofectamin-2000 (Invitrogen) according to manufacturer’s instruction as previously described^24^.

### Western blotting

Before and after treatments with the indicated drugs, or transfections with the siRNAs against indicated genes, cell pellets were lysed and Western blotting was performed as previously described^25^. The antibodies used to detect the proteins in this study were described above.

### Immunofluorescence

FANCD2 foci, γH2AX foci and RAD51 foci were determined by immunofluorescence assay as previously described^25^. Primary antibodies were mouse monoclonal anti-FANCD2 and anti-phospho-ser139 H2AX, and rabbit polyclonal anti-RAD51. Secondary antibodies were anti-mouse IgG.

### Alkaline comet assay

The modified alkaline comet assay was performed to detect DNA damages (SSB and DSB form) as previously described^26^. After slides were neutralized in 0.4 M tris-HC1 and stained with ethidium bromide, five hundred randomly chosen cells per slide were captured using a fluorescence microscope (Olympus IX71), and mean tail moment was quantified using Comet Score version 1.5 (Tri Tek).

### Cell apoptosis and cell cycle analysis

The percentage of apoptosis cells was determined before and after treatment with the indicated drugs or transfection with siRNAs against indicated genes, using flow cytometry with Annexin V-FITC/PI staining, as previously described^26^. Cell cycle analysis was performed using propidium iodide stain and flow cytometry, as previously described^25^.

### Statistical analysis

All data were expressed as the mean ± standard deviation of at least three independent experiments. The IC_50_ was calculated as the gemcitabine concentration that kills 50% of cells of untreated control. Statistical analyses were carried out using Student *t* test or one-way ANOVA with a Tukey’s post-hoc test by SPSS18.00 version (SPSS Inc., Chicago,II). P-values < 0.05 were considered significant.

## Results

### Depletion of the FA pathway factors sensitized LSC cells to gemcitabine

**Figure 1:**
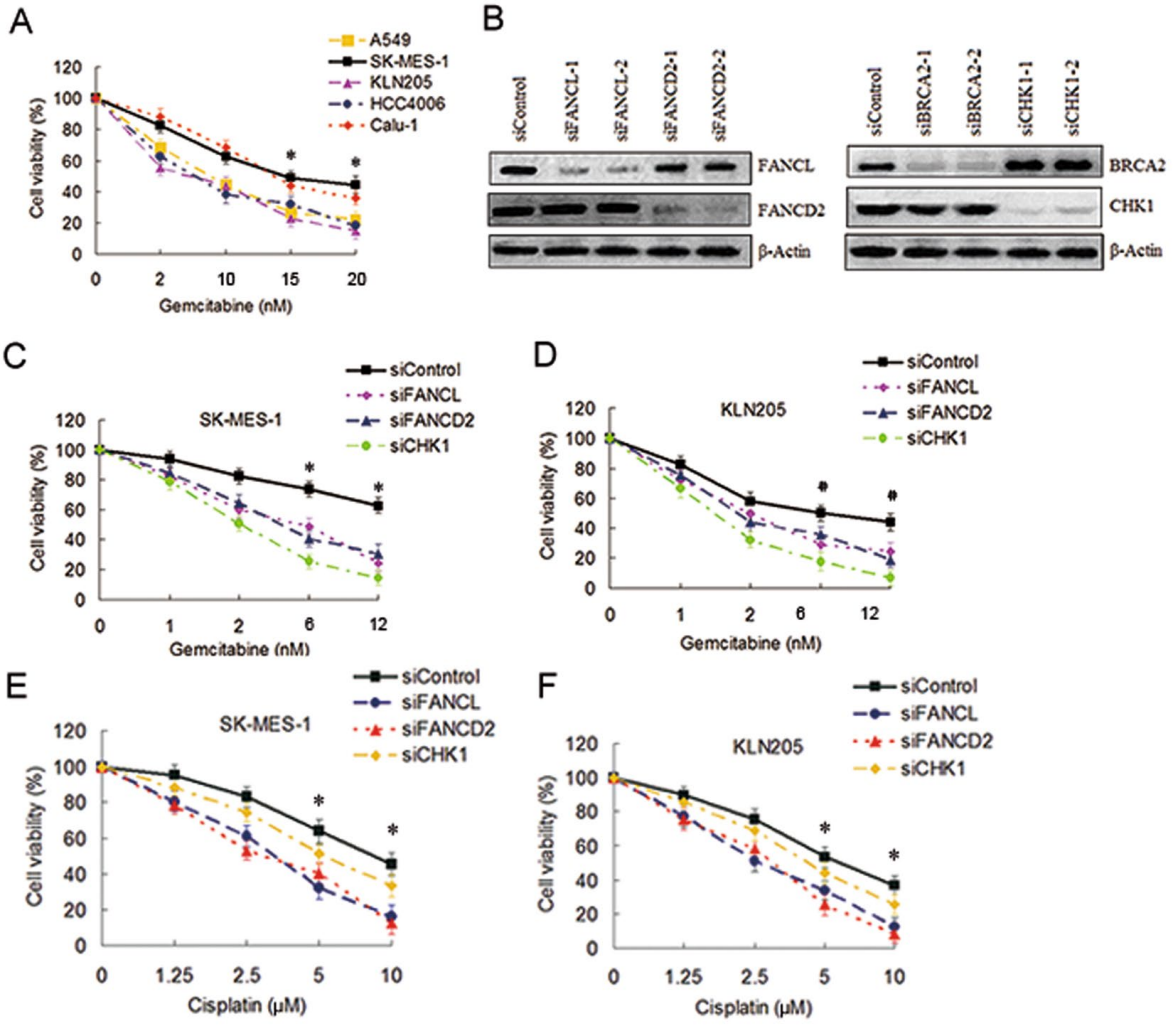
Depletions of the FA pathway factors increased the sensitivity of gemcitabine to SK-MES-1 cells. (**A**) A549, SK-MES-1, KLN205, HCC4006 and Calu-1 cell lines growing in 96-well plates were treated with gemcitabine at the indicated dose for 4 h. The CCK-8 assay was used to determine cell viability (^*^SK-MES-1 or Calu-1 as compared with A549, KLN205, and HCC4006 respectively, *P* < 0.05). (**B**) Western blot of FANCL-, FANCD2-, BRCA2- and CHK1-depleted SK-MES-1 cells to verify the efficiency of the siRNA transfections. β-actin was used as loading control. Uncropped images are presented in Supplementary Figure S5. (**C**) SK-MES-1 and (**D**) KLN205 cells growing in 96-well plates were transfected with various siRNAs as indicated. Cell viability was detected by the CCK-8 assay following gemcitabine treatment for 4 h (^*^siContrl as compared with siFANCL and siFANCD2, *P* < 0.005, compared with siCHK1, *P* < 0.001; ^#^siControl as compared with siFANCL and siFANCD2, *P* < 0.05, compared with siCHK1, *P* < 0.005). (**E**) SK-MES-1 and (**F**) KLN205 cells growing 96-well plates were transfected with various siRNA as indicated. Cell survival was measured by the CCK-8 assay following cisplatin treatment (siControl as compared with siFANCL and siFANCD2, *P < 0.01).

Previous studies have reported that CHK1 inhibition sensitized cancer cells to gemcitabine^19–21^, silencing of the FA Pathway genes enhanced cytotoxicity of cisplatin to lung cancer cells^24,26^. But little has been known about the impact of the FA pathway suppression on the sensitivity of gemcitabine to NSCLC cells. In this study, firstly we assess the sensitivity of various NSCLC cell lines to gemcitabine. As shown in Fig. 1A, SK-MES-1 and Calu-1 cell lines were more resistant to gemcitabine than A549, KLN205 and HCC4006 cell lines. Because gemcitabine in combination with cisplatin is preferred for the treatment of LSC, we chose two LSC cell lines SK-MES-1 and KLN205 as the research object in subsequent experiments. The former is relative resistant to gemcitabine (IC_50_: 20.56 ± 6.83), the latter is more sensitive to gemcitabine (IC_50_: 8.56 ± 3.45). To address whether disabling the FA pathway can influence the sensitivity of the LSC cells to gemcitabine, we initially used siRNA transfection approaches to deplete CHK1 and the FA pathway factors, such as FANCL, FANCD2 and BRCA2 (Fig. 1B) in SK-MES-1 and KLN205 cell lines. The cell viability assay showed that depletion of FANCL and FANCD2 can sensitize the two LSC cell lines to gemcitabine, although the degree of sensitization was infeior to CHK1 silencing (Fig. 1C,D). It is noteworthy that the sensitization effect by depleting FANCL, FANCD2 or CHK1 in SK-MES-1 cells was more remarkable than in LKN205 cells, for instance, the IC_50_ of gemcitabine in the SK-MEK-1 cells decreased from 20.56 ± 6.83 to 5.14 ± 2.27 and 2.86 ± 0.78 after FANCD2 and CHK1 depletion respectively, whereas the IC_50_ in the KLN205 cells decreased from 8.56 ± 3.45 to 3.77 ± 1.52, and 1.85 ± 0.39 with same treatment, respectively (Fig. 1C,D). In the other hand, the degree of sensitization to cisplatin by depleting the FA pathway factors was more significant than that by silencing CHK1 (Fig. 1E,F). These results suggest that the FA pathway and CHK1 are implicated in gemcitabine resistance in SK-MES-1 cells.

### Depletion of the FA pathway factors increased sensitivity of LSC cells to CHK1 inhibitor

**Figure 2:**
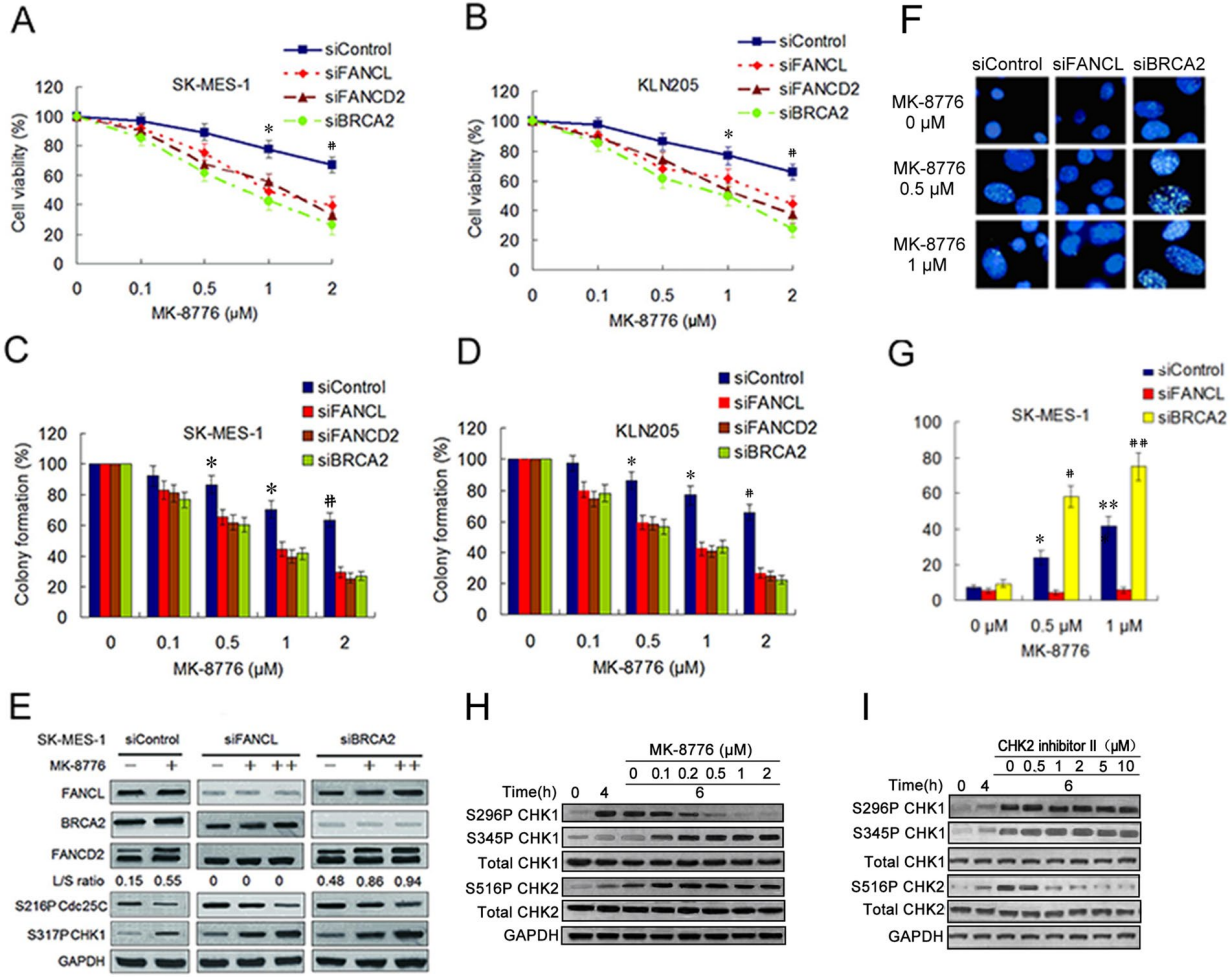
Depletions of the FA pathway factors sensitized LSC cells to MK-8776. (**A**,**B**) The viability analysis were performed using the CCK-8 assay in SK-MES-1 and KLN205 cell lines depleted of the FA pathway factors after MK-8776 treatment for 6 h. (siControl as compared to siFANCL, siFANCD2 and siBRCA2, *P < 0.05; ^#^ *P* < 0.01). (**C**,**D**) The two LSC cell lines were treated with MK-8776 following transfection with various siRNA as indicated. The cells were stained by crystal violet and total colonies were counted after two weeks. Colony numbers of control-treated cells were set as 100%. (siControl as compared to siFANCL, siFANCD2 and siBRCA2, ^*^ *P* < 0.01; ^#^ *P* < 0.005). (**E**) Western blotting detecting monoubiquitination of FANCD2 and phosphorylation of Cdc25C (S216) and CHK1 (S317) in SK-MES-1 cells transfected with siControl, siFANCD2 and siBRCA2. The cells were either treated with MK-8776 (0.5 μM+ , 1 μM++) or DMSO control for 6 h. GAPDH was used as loading control. Uncropped images are presented in Supplementary Figure S6. (**F**) SK-MES-1 cells were treated with MK-8776 at the indicated doses for 6 h following transfection with siFANCL or siBRCA2, and fixed and immunostained using anti-FANCD2 antibody. (**G**) The percentage of cells with >20 FANCD2 foci was quantified using Spot Advanced RT software (the cells treated with 0.5 μM MK-8776 as compared to the cells treated with 0 μM MK-8776, ^*^ *P* < 0.05, ^#^ *P* < 0.01; the cells treated with 1 μM MK-8776 compared to the cells treated with 0 μM MK-8776, ^**^ *P* < 0.01, ^##^ *P* < 0.001). (**H** and **I**) SK-MES-1 cells were treated with 5 nM gemcitabine for 4 hours. The drug was then replaced with MK-8776 or CHK2 inhibitor II at the indicated doses and the cells harvested after an additional 6 hours. Cell lysates were analyzed by Western blotting for the indicated protein.

FA pathway deficient cells acquire DNA damage in S phase and are accumulated in the G2 phase of the cell cycle. This G2 phase accumulation correlates with the activation of a G2/M checkpoint^27^, which at least partly comes from hyperactive CHK1 activity. The CHK1 hyperactivity may serve as a compensatory mechanism for the FA pathway deficiency. Thus we infer that the cancer cells depleted of the FA pathway factors are hypersensitivity to CHK1 inhibition. To examine this inference, we assess the effect of depletion of the FA pathway factors on the sensitivity of selective CHK1 inhibitor MK-8776 in SK-MES-1 and KLN205 cell lines. The two LSC cell lines were incubated with MK-8776 at increasing concentration for 6 h following transfections with siFANCL, siFANCD2 or siBRCA2. As shown in Fig. 2A,B, the two cell lines depleting FANCL, FANCD2 or BRCA2 were more sensitive to MK-8776 compared to the cells transfected with control siRNA. Similar results were observed in colony formation assay (Fig. 2C and D).

To better characterize the molecular nature of the FA pathway and CHK1 interaction, we evaluate the relationship between the CHK1 activity and the function status of the FA pathway in the two LSC cell lines. Results showed that silencing of FANCL and BRCA2 increased phosphorylations of Cdc25C (S216) and CHK1 (S317), indicating that CHK1 was activated by inhibiting the FA pathway (Fig. 2E, Supplementary Fig. S1A). Addition of MK8776 inhibited the phosphorylation of Cdc25C, implying abrogation of CHK1 function in the cells with the FA pathway deficiency. Additionally, CHK1 phosphorylation was further increased by MK-8776 treatment, suggesting enhanced ATR activity, which is consistent with previous studies demonstrating an increase in ATR-mediated phosphorylation of CHK1 following the inhibition of CHK1 activity^28^. Meanwhile, MK-8776 treatment elevated the levels of FANCD2 monoubiquitination in the two cell lines transfected with control siRNA and siBRCA2 which functions in downstream of the FA pathway (Fig. 2E, Supplementary Fig. S1A), in parallel with an increase of the percentage of cells with FANCD2 foci (Fig. 2F,G, Supplementary Fig. S1B,C), indicating that the FA pathway is activated after suppression of CHK1 activity. The findings imply that the CHK activity and the FA pathway function in compensatory manner to repair DNA damage and maintain genome integrity.

When cells are treated with CHK1 inhibitor, CHK1 is phosphorylated at S345 by ATR, whereas phosphorylation at S296 is a consequence of autophosphorylation such that treatment of DNA damage cells with CHK1 inhibitor inhibits only autophosphorylation. To determine the concentration of MK-8776 that inhibits CHK1, we examined CHK1 phosphorylation at S296 and S345 after sequential treatment with gemcitabine and MK-8776. Gemcitabine-damaged SK-MES-1 cells were treated with 0 to 2 μM MK-8776 for 6 hours. Significant reduction in S296 phosphorylation was observed at and above 0.5 μM while phosphorylation at S345 actually increased (Fig. 2G). CHK2 is also activated as a consequence of DNA damage and phospho-S516 reflects autophosphorylation^29^. There was no inhibition of CHK2 phosphorylation on S516 at concentration as high as 1 μM MK-8776 (Fig. 2G), which is consistent with its selectivity for CHK1 over CHK2^30^. As a control, we also treated the cells with the CHK2 inhibitor II (a 2-arylbenzimidazole-based and high selective CHK2 inhibitor)^31^, which showed marked inhibition of S516-CHK2 at 1 μM but has no impact on phospho-CHK1. Therefore, we used 0.5 or 1 μM MK-8776 in the following experiment.

### The effect of co-inhibition of the FA pathway and CHK1 on DDR and the accumulation of DNA damage induced by gemcitabine

**Figure 3:**
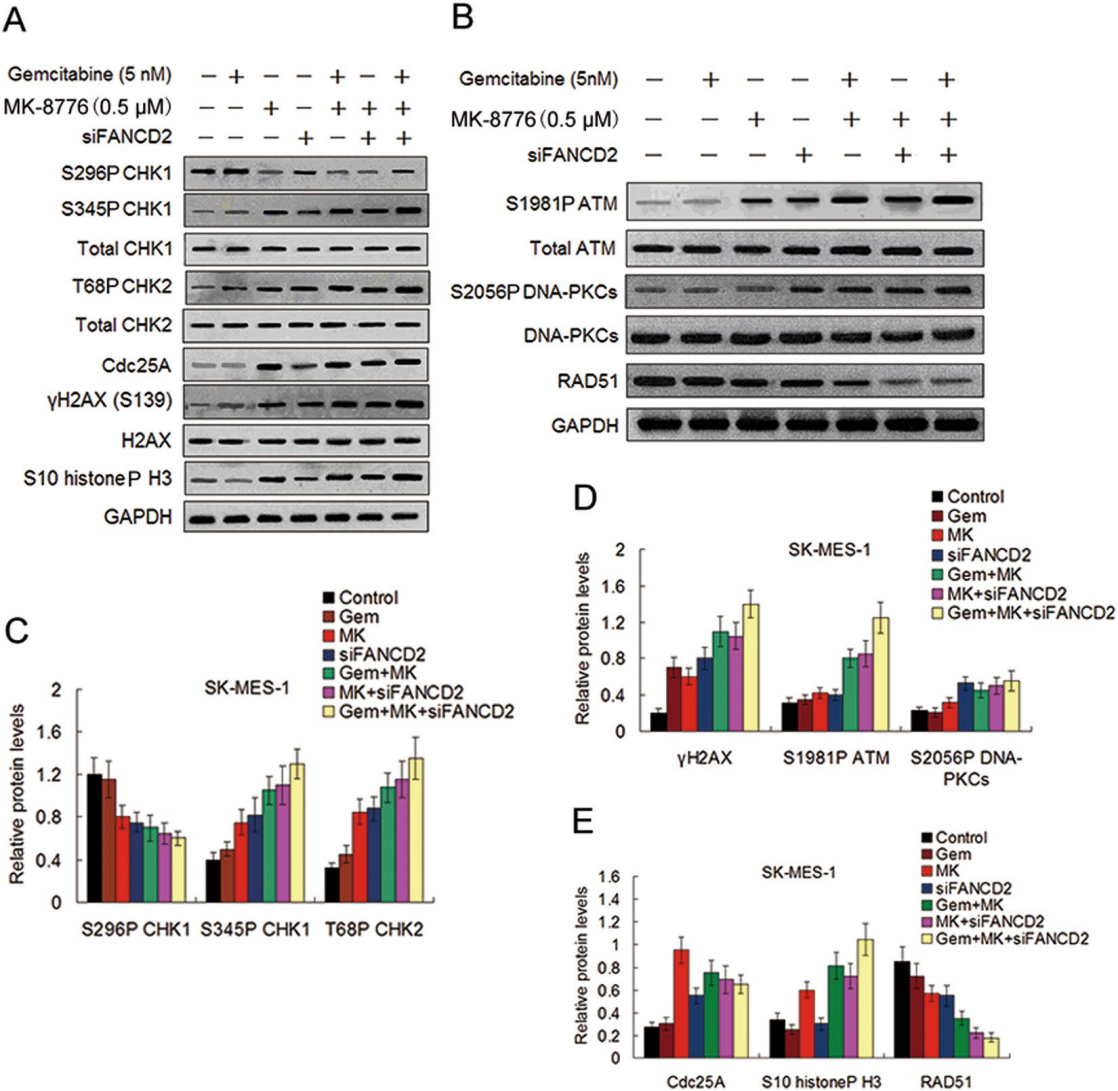
Effect of MK-8776 and FANCD2 knockdown on gemcitabine-induced phosphorylation and expression of proteins involved in the cell DNA damage checkpoint pathways. (**A**,**B**) Before and after siFANCD2 transfection, the SK-MES-1cells were treated with gemcitabine for 4 h, or MK-8776 for 6 h, or co-treated with gemcitabine and MK-8776 for 4 h and then with MK-8776 alone for another 6 h after removal of gemcitabine. Cell lysates were analyzed by Western blotting for the indicated proteins as described in the materials and methods, GAPDH was used as a loading control. Uncropped images are presented in Supplementary Figure S7A and B. (**C**) The intensity of S296P CHK1, S345P CHK1, T68P CHK2, and (**D**) γH2AX, S1981P ATM, and S2056P DNA-PKCs bands shown in (**A**,**B**) was quantified by densitometry and normalized against that of their nonphosphorylated bands. (**E**) The intensity of Cdc25A, S10 histoneP H3, and RAD51 bands was quantified by densitometry and normalized against that of the GAPDH bands.

To evaluate the impact of co-inhibition of the FA pathway and CHK1 on gemcitabine-caused activation of the DDR pathways, we test the effect of FANCD2 silencing and CHK1 inhibitor MK-8776 on phosphorylation levels of CHK1, CHK2, and H2AX in SK-MES-1 and KLN205 cell lines in the presence and absence of gemcitabine. Western blot assay showed that phosphorylation of CHK1 (S345) and H2AX (S139) were increased in response to either gemcitabine or MK-8776 in line with activation of the DNA damage response pathway, while the combination of the two resulted in further increase in S345P CHK1 (Fig. 3A,C and D, Supplementary Fig. S2A,C and D), which have been attributed to disruption of PP2A-mediated dephosphorylation on this site and increased DNA damage that accumulate when CHK1 cannot regulate replication^32^. Similarly, the combination of gemcitabine and MK-8776 produced a greater level of T68P CHK2. As expected, CHK1 autophosphorylation (S296P) was inhibited by MK-8776 both in the presence and absence of gemcitabine, indicating that CHK1 activity was suppressed by MK-8776. Consistent with CHK1 activity being inhibited by MK-8776, the decrease of Cdc25A expression induced by gemcitabine was inhibited by MK-8776 (Fig. 3A,E and Supplementary Fig. S2A,E). In addition, MK-8776 alone induced an increase in phosphorylated histone H3 (S10), which is a marker of mitosis, suggesting that cell mitotic entry was initiated^16^. The combination of gemcitabine and MK-8776 further increased the expression of phosphorylated histone H3, indicating the abrogation of gemcitabine-mediated cell cycle arrest by MK-8776 and further increase of mitotic entry. Moreover, we found that the cells depleted of FANCD2 exhibited induction of phosphorylation of CHK1 (S345), CHK2 (T68), and H2AX (S139). Importantly, these increased phosphorylations in depleted FANCD2 cells reached the maximal levels in response to the treatment of gemcitabine plus MK-8776 (Fig. 3A,C and D, Supplementary Fig. S2A,C and D), implying that co-inhibition of the FA pathway and CHK1 significantly increased DDR and the accumulation of DNA damage caused by gemcitabine.

In parallel analysis, we also found that inductions of phosphorylations of ATM (S1981) and DNA-PKCs (S2056) were in general agreement with γH2A expression in response to MK-8776, or MK-8776 plus gemcitabine or MK-8776 plus FANCD2 knockdown (Fig. 3B,D and Supplementary Fig. S2B,D), suggesting that these LSC cells with CHK1 inhibition were defective in repair of DNA damage and rely on compensatory activation of the ATM pathway^33^. These results are coincident with previous studies demonstrating that ATM and DNA-PKCs contribute to γH2A induction by CHK1 inhibition following replication stress in human cancer cells^34^.

### Depletion of FANCD2 combined with CHK1 inhibitor markedly potentiated cytotoxicity of gemcitabine to LSC cells

**Figure 4:**
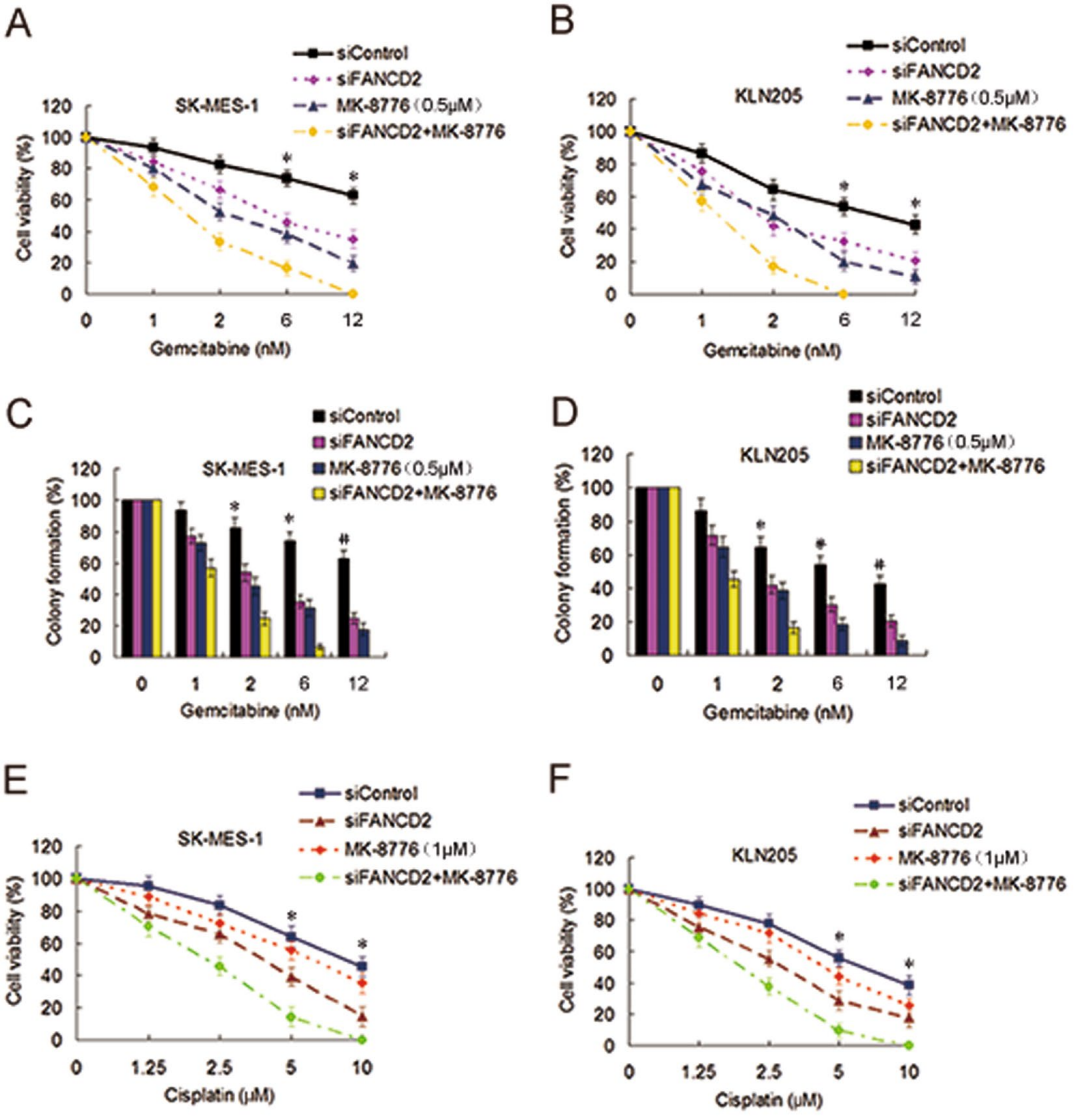
Depletion of FANCD2 synergized with MK-8776 to enhance cytotoxicity of gemcitabine to LSC cells. (**A**) Before and after siFANCD2 transfection, SK-MES-1 cells and (**B**) KLN205 cells were treated with gemcitabine at different doses for 4 h, or MK-8776 for 6 h, or co-treated with gemcitabine and MK-8776 for 4 h and then with MK-8776 alone for another 6 h after removal of gemcitabine. The cell viability was detected by the CCK-8 assay. (siControl as compared to siFANCD2 and MK-8776, *P < 0.01; siControl as compared to siFANCD2 plus MK-8776, *P < 0.001). (**C**,**D**) The two LSC cell lines were treated with the methods as described above, and then cells were stained by crystal violet. Total colonies were counted after two weeks. Colony numbers of control cells were set as 100% (siControl as compared to siFANCD2 and MK-8776, *P < 0.01; siControl as compared to siFANCD2 plus MK-8776, ^*^ *P* < 0.001; siControl as compared to siFANCD2 and MK-8776, ^#^ *P* < 0.01). (**E**,**F**) The two LSC cell lines were treated with the methods as described above, and the cell viability was determined by the CCK-8 assay following cisplatin treatment at various doses (siControl as compared to siFANCD2, *P < 0.01; siControl as compared to siFANCD2 plus MK-8776, *P < 0.001).

Because the FA pathway and CHK1 mutually compensate in repairing DNA damage and maintaining genome integrity, we reason that co-inhibition of CHK1 and the FA pathway can produce more profound sensitization of the LSC cells to gemcitabine. Indeed, FANCD2 knockdown combined with MK-8776 treatment further potentiated killing effect of gemcitabine to the two LSC cell lines (Fig. 4A–D), compared to individual FANCD2 knockdown or MK-8776 treatment, indicating that FANCD2 depletion has an enhancement effect with CHK1 inhibition in sensitizing the LSC cells to gemcitabine. In addition, the two LSC cell lines co-treated with FANCD2 knockdown and MK-8776 were more sensitive to cisplatin than those treated with FANCD2 knockdown or MK-8776 alone (Fig. 4E,F and Supplementary Fig. S3A,B), demonstrating that co-inhibition of the FA pathway and CHK1 also produce an enhancement effect in increasing sensitivity of cisplatin to the LSC cells, although MK-8776 alone did not sensitize the cells to cisplatin (Fig. 4E,F).

**Figure 5:**
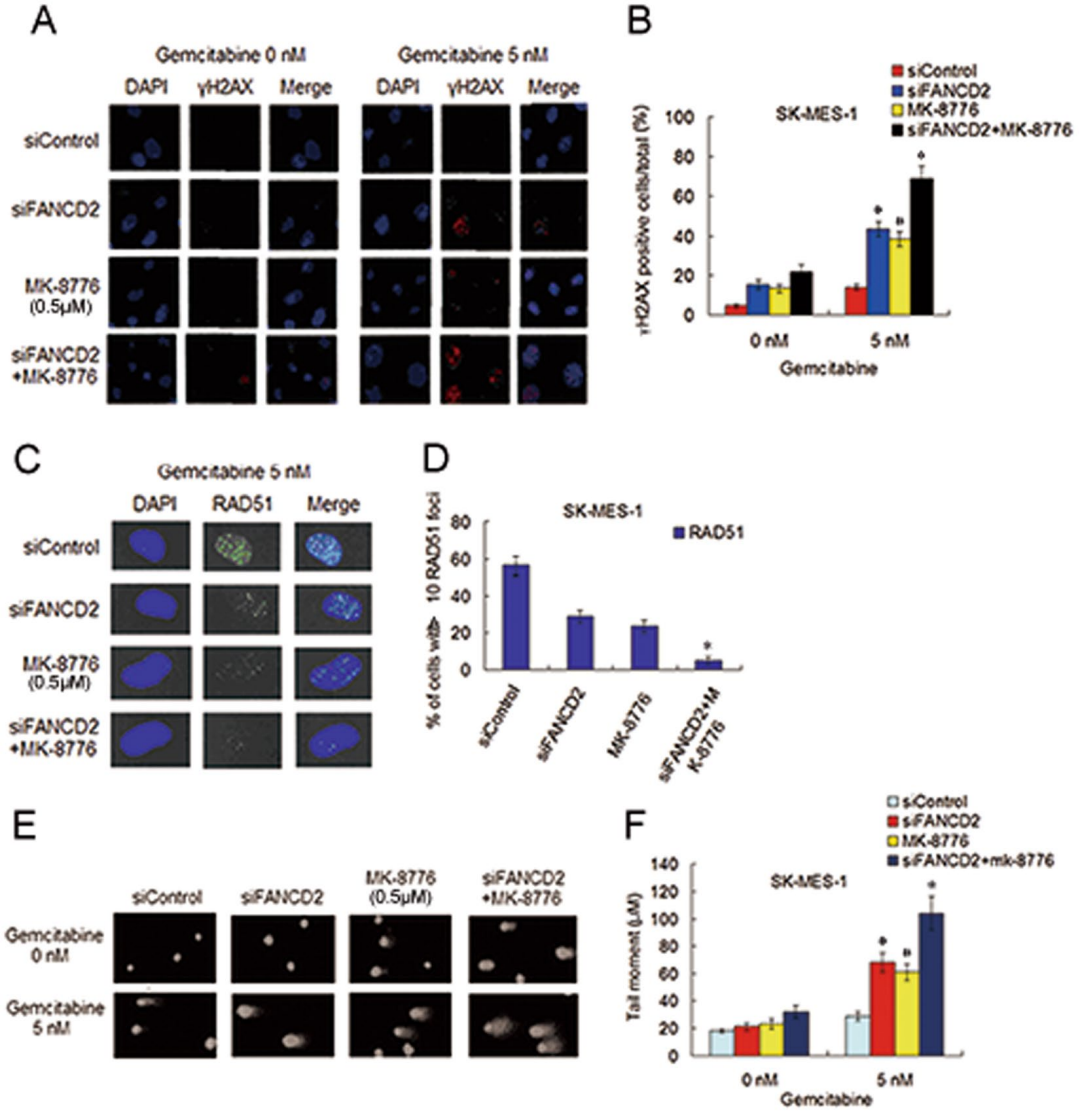
Depletion of FANCD2 plus MK-8776 treatment in SK-MK-1 cells led to marked accumulation of DNA damage induced by gemcitabine. (**A**) Before and after transfection with siFANCD2, SK-MES-1 cells were treated with gemcitabine or MK-8776 alone, or gemcitabine plus MK-8776, culture in fresh medium for another 48 h, fixed and immunostained with an anti-γH2AX antibody. (**B**) The percentage of γH2AX foci positive cells was quantified using Matafer Software (siFANCD2 plus MK-8776 as compared with siControl, *P < 0.001; compared with siFANCD2 or MK-8776, *P < 0.05; siFANCD2 and MK-8776 as compared with siControl, ^#^ *P* < 0.01). (**C**,**D**) Before and after transfection with siFANCD2, cells were treated with methods as described above, and fixed and immunostained with RAD51 antibody. The percentage of cells with >10 RAD51 foci was quantified from Image Software (siFANCD2 plus MK-8776 as compared with siControl, *P < 0.001; compared with siFANCD2 or MK-8776, *P < 0.01). (**E**,**F**) Before and after transfection with siFANCD2, SK-MES-1 cells were treated with gemcitabine or MK-8776 alone, or gemcitabine plus MK-8776. Alkaline comet assay was used to determine SSBs and DSBs, and the images show detectable comet tail when visualized under a fluorescent microscope. Tail moment in the cells were quantified using Comet Score Software version 1.5 (siFANCD2 plus MK-8776 as compared with siControl, *P < 0.001; compared with siFANCD2 or MK-8776, *P < 0.05; siFANCD2 and MK-8776 as compared with siControl, ^#^ *P* < 0.01).

To determine whether gemcitabine chemosensitization via co-inhibition of CHK1 and the FA pathway is attribute to the suppression of DSBs repair by RAD51-mediated HHR and crosslink repair by the FA pathway, we evaluate the repair ability of DNA damage caused by gemcitabine in the two cell lines by determining the formation of γH2AX foci which is an sensitive marker of DSBs^35^, and the assembly of RAD51 foci at the site of DSBs, and the length of tail moment in the comet assay that reflect the degree and presence of single strand breaks (SSDs) and DSBs. The results showed that after gemcitabine exposure, the cells treated with FANCD2 depletion plus MK-8776 display an significantly increased the percentage of γH2AX foci positive cells and a significantly decreased the number of the cells with >10 RAD51 foci, as well as a prominently increased tail moment, when compared with the cells treated with FANCD2 knockdown or MK-8776 alone (Fig. 5A–F, and Supplementary Fig. S3C–H). Taken together, these data indicate that the marked impediment of the repair of gemcitabine-induced DNA damage after co-inhibition of CHK1 and the FA pathway is responsible for the strong gemcitabine chemosensitization.

### Depletion of FANCD2 combined with MK-8776 notably facilitated caspase-3 dependent apoptosis induced by gemcitabine in the LSC cells

**Figure 6:**
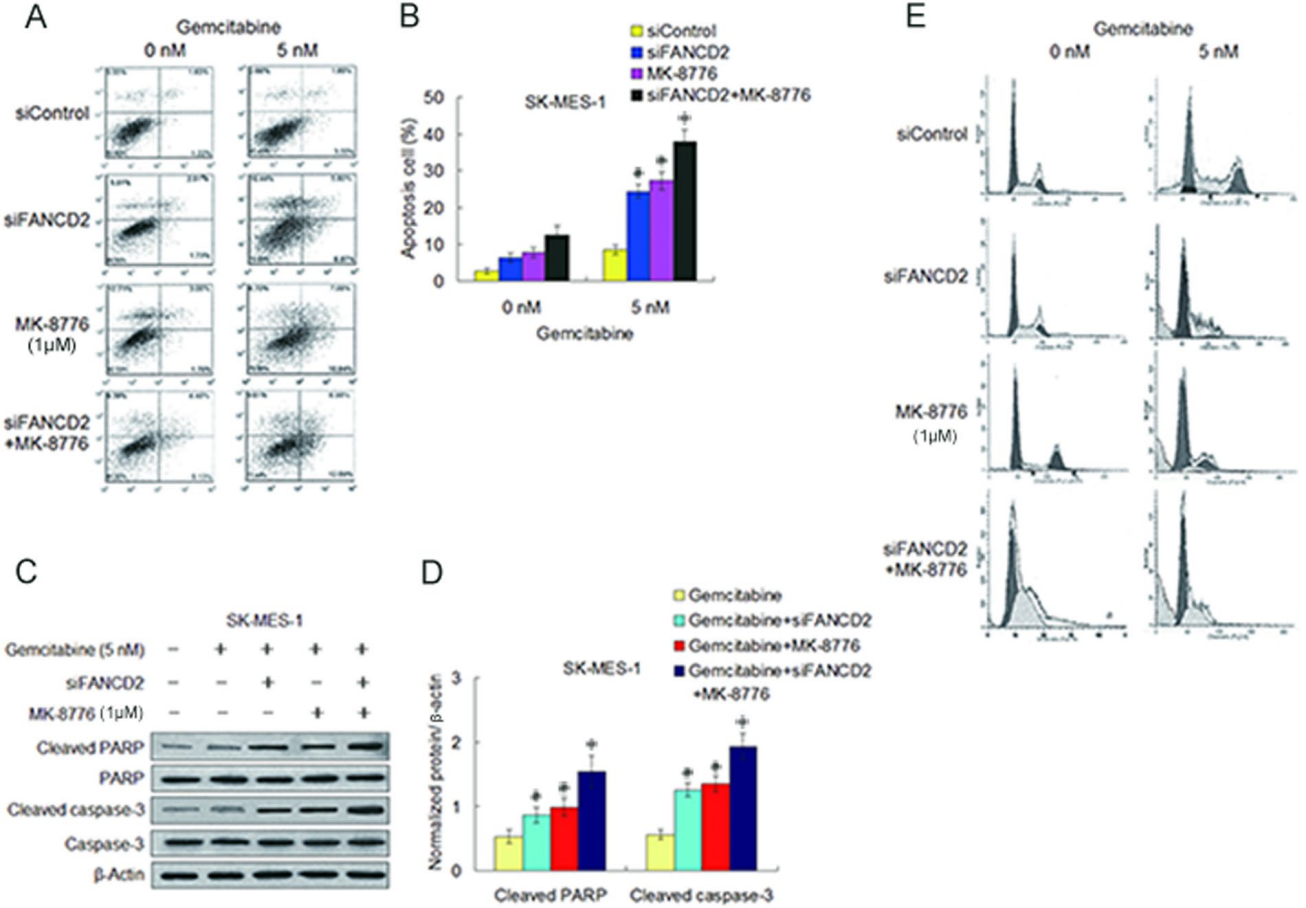
Depletion of FANCD2 combined with MK-8776 result in significant increase of caspase-3 dependent apoptosis caused by gemcitabine. (**A**,**B**) Before and after transfection with siFANCD2, SK-MES-1 cells were treated with gemcitabine, or MK-8776, or gemcitabine plus MK-8776. Cell apoptosis was detected by flow cytometry with Annexin γ-FITCIPI staining, and apoptosis rates were quantified by computer software (siFANCD2 plus MK-8776 as compared with siControl, *P < 0.001; as compared with siFANCD2 or MK-8776, *P < 0.05; siFANCD2 or MK-8776 as compared with siControl, ^#^ *P* < 0.01). (**C**,**D**) Cleaved PARP and cleaved caspase-3 were determined by Western blotting in SK-MES-1 cells transfected with siFANCD2, or siFANCD2 transfection plus gemcitabine, or gemcitabine plus MK-8776, or siFANCD2 transfection combined with gemcitabine plus MK-8776. β-actin was used as loading control. Uncropped images are presented in Supplementary Figure S8. (gemcitabine plus MK-8776 plus siFANCD2 as compared with gemcitabine alone, *P < 0.005; compared with gemcitabine plus siFANCD2 or gemcitabine plus MK-8776, *P < 0.05; gemcitabine plus siFANCD2 or gemcitabine plus MK-8776 as compared with gemcitabine alone, ^#^ *P* < 0.05). (**E**) Representative imagines of cell cycle analyses showed that MK-8776 reduced the S phase accumulations induced by gemcitabine, and co-treatment with MK-8776 and siFANCD2 transfection increased sub-G1 population simultaneous with decrease of S phase accumulation induced by gemcitabine in SK-MES-1 cells.

Finally we examined whether the enhancement of gemcitabine-produced tumoricidal effect by co-inhibition of CHK1 and the FA pathway is associated with apoptosis in the two LSC cell lines. In coincidence with the results in cell viability measurement, FANCD2 knockdown combined with MK-8776 notably increased the percentage of apoptosis cells produced by gemcitabine compared to FANCD2 knockdown and MK-8776 treatment alone (Fig. 6A,B, Supplementary Fig. S4A, B). Moreover, co-treatment with FANCD2 knockdown plus MK-8776 clearly increased the levels of cleaved caspase-3 and PARP proteins in the two LSC cell lines when compared to individual treatment with FANCD2 knockdown or MK-8776 (Fig. 6C,D, Supplementary Fig. S4C,D), which are identical with studies showing that cleaved caspase-3 is a marker of chemosensitization by CHK1 inhibitor^36^. Additionally, cell cycle analysis showed that gemcitabine induced the arrest of G1 and S phases in the LSC cells, and addition of MK-8776 depress S phase arrest induced by gemcitabine. Importantly, cells treated with gemcitabine plus MK-8776 following depletion of FANCD2 appeared obvious sub-G1 population simultaneous with inhibition of S phase accumulation (Fig. 6E, Supplementary Fig. S4E), suggesting that inhibition of CHK1 in gemcitabine-treated cells cause abrogation of S phase arrest and induce premature entry of S phase cells into mitosis^34,37^ that may lead to the phenomenon of mitotic catastrophe, which is considered a lethal event that progresses to apoptosis^38^. Suppression of the FA pathway may promote this process. This data represent another independent verification of the enhancement effect between CHK1 inactivation and the FA pathway suppression in sensitizing the LSC cells to gemcitabine.

## Discussion

Studies have shown that CHK1 inhibitors can enhance the cytotoxicity of gemcitabine to cancer cells by abrogating inhibition of replication origin firing, destabilizing stalled replication forks, and inducing mitotic death^19–21,34^. This study showed that depletion of the FA pathway factors also sensitized LSC cells to gemcitabine. Moreover, the sensitization effect to gemcitabine by silencing the FA pathway factors was more significant in SK-MES-1, a gemcitabine relative resistant LSC cell line, than KLN205 cells which are more sensitive to gemcitabine, suggesting that the FA pathway is involved in gemcitabine resistant. Meanwhile, we found that suppression of the FA pathway sensitized the LCS cells to CHK inhibitor MK-8776. This imply that defect of the FA pathway activate compensatory DNA repair mechanisms including CHK1 mediated G2/M checkpoint, which is supported by evidence that depletion of FANCL and BRCA2 elevated phosphorylation levels of CHK1 (S317) that indicating an enhanced CHK1 activity^39^. And CHK1 inhibition led to the up-regulation of the FA pathway which is reflected by the increases of FANCD2 monoubiquitination levels and foci formation in the FA pathway upstream proficient cells. The data indicate that the LSC cells with the FA pathway deficiency are hyper-dependent on the ATR/CHK1 pathway for survival, while the cells deficient in CHK1 function have to rely on the FA pathway to maintain genome stability.

In the light of these findings, we speculate that co-inhibition of the FA pathway and CHK1 can significantly potentiate killing effect of gemcitabine to the LSC cells. We found that depletion of FANCD2 or MK-8776 treatment alone moderately sensitized SK-MES-1 and KLN205 cells to gemcitabine. Furthermore, the depletion of FANCD2 combined with MK-8776 further increased the sensitivity of the two LSC cell lines to gemcitabine as compared to individual treatment with FANCD2 depletion or MK-8776. Our results clearly show that simultaneous suppressions of the FA pathway and CHK1 abrogate the G2/M checkpoint response and block the FA pathway DNA repair and result in synthetic lethal effects to DNA damage caused by gemcitabine. The data support the idea that the FA pathway and CHK1 are functionally compensatory in the repair of DNA damage, and inactivation of one of the two pathways lead to DNA damage accumulation, triggering compensatory activation of other pathway, co-inactivation of the two pathways result in dramatically enhanced cell killing effect induced by genotoxic agents when compared to inactivation of any one pathway^40^.

The main mechanism of tumoricidal effect of gemcitabine is inhibition of ribonucleotide reductase and DNA elongation by stalling replication fork progression^41^. Prolonged replication stalling lead to replication fork collapse and the generation of replication-dependent DNA DSBs^42^, which are the most lethal lesions induced by genotoxic drugs, and mainly repaired through two pathways HHR and non-homologous end-joining (NHEJ). CHK1 is now recognized as having additional roles in replication fork stability, replication origin firing and HHR^34,43,44^, and it is the latter of these roles that appears important for the efficacy of combination of gemcitabine and CHK1 inhibitor. CHK1 participate in HHR by phosphorylating BRCA2 which interacts with and recruits RAD51 to single-strand DNA, and also directly phosphorylating RAD51 which is also required for recruitment of RAD51 to the site of DNA damage^43^. The FA pathway regulates cellular response to replication stress and plays a key role in the repair of DNA crosslinks and DSBs in coordination with the HHR pathway^22^. Our results showed that gemcitabine in combination with CHK1 inhibitor resulted in dramatic increase of DNA damage as evidenced by the prolongation of tail moment in the comet assay, ATR-mediated γH2AX induction, and the activation of the ATR/CHK1 and ATM/CHK2 pathways. Meantime, an increased cell mitotic entry was observed after treatment of gemcitabine plus MK-8776, which was reflected by elevated phosphorylation of histone H3 (S10)^45^. Similarly, combination of FANCD2 depletion and gemcitabine cause an increase of unrepair DNA SSBs and DSBs. Furthermore, when FANCD2 knockdown was combined with gemcitabine, addition of MK-8776 yielded more profound DDR and more severe impediment of DNA repair which were in consistent with reduction in cell viability, demonstrating that the strong gemcitabine-chemosensitization produced by co-inhibition of the FA pathway and CHK1 mainly result from grievous block of repair of gemcitabine-caused DNA damage.

It is well known that chemotherapeutic agents such as gemcitabine impart to DNA and consequently activate cell cycle checkpoints resulting in cell cycle arrest. MK-8776 showed checkpoint abrogating activity when combined with gemcitabine which was reflected by the increase of phosphorylated histone H3, a marker of entry into mitosis^45^. Induction of Cdc25A phosphatase after the combination treatment further confirmed mitotic progression. Cell cycle progression into mitosis with damaged DNA results in increased cell death as indicated by an increase of caspase-3 dependent apoptosis. The effect was further enhanced by the FA pathway inhibition via FANCD2 knockdown. Although there is still some controversy about the exact mechanism of increased gemcitabine cytotoxicity by suppressing CHK1^32^, our data provide evidence that a great degree of DNA damage due to checkpoint abrogation, lack of DNA repair, and subsequent mitosis catastrophe is responsible for the sensitization of the LSC cells to gemcitabine.

An interesting finding in this study is that MK-8776 also exhibited an enhancement effect with FANCD2 knockdown in increasing antitumor efficacy of cisplatin to LSC cells. Our previous studies have demonstrated that silencing the FA pathway factors sensitize NSCLC cells to cisplatin^24,26^. While MK-8776 alone causes little change to chemosensitivity of cisplatin, it combined with FANCD2 depletion resulted in synthetic lethal interaction in response to cisplatin. The results are particularly significant because combination of cisplatin with gemcitabine is first-line chemotherapy for advanced LSC patients with the wild-types of EGFR and ALK^6^. Thus simultaneous sensitization to gemcitabine and cisplatin by co-inhibiting CHK1 and the FA pathway will surely bring forth good therapeutic efficiency.

It has been reported that some cell lines are hypersensitive to CHK1 inhibitor MK-8776 alone, with induction of γH2AX and DNA DSBs within several hours^46,47^. Therefore, MK-8776 can be considered a genotoxic agent that induce genomic instability and cell death, although it was often used as a sensitizer to sensitize cancer cell to chemotherapeutic agents and radiation *in vivo* and *in vitro* ^19,20,46^. Therapeutic inhibitors of the FA pathway are already in development^48^, further investigations for assessing the potential efficacy of these drug combinations are required in future preclinical and clinical studies.

In conclusion, this study demonstrates that cytotoxicity of gemcitabine can be significantly enhanced by co-inhibiting CHK1 and the FA pathway in LSC cells. A compensatory relationship is observed between the CHK1 pathway and the FA pathway in response to gemcitabine-induced DNA damage that results in hyper-activation of one pathway when other is inactivated. CHK1 inhibitor MK-8776 combined with FANCD2 knockdown lead to synthetic lethality following gemcitabine treatment. This study provide additional evidence for developing personalized therapeutic strategies in treatment of advanced LSC with combination of standard chemotherapy and inhibitors of CHK1 and the FA pathway.

## Electronic supplementary material

Supplementary figures and figure legends
