# Supplementary material for: Suppression of the FA pathway combined with CHK1 inhibitor hypersensitize lung cancer cells to gemcitabine
Source: Sci Rep. 2017 Nov 8;7:15031. doi: 10.1038/s41598-017-15172-4 (PMC5678185; doi:10.1038/s41598-017-15172-4)
Supplement: Supplementary file 1 — Supplementary figures and figure legends [file 41598_2017_15172_MOESM1_ESM.pdf]

Supplementary information

Suppression of the FA pathway combined with CHK1 inhibitor hypersensitize lung cancer cells to gemcitabine

Chun-Hau Dai[1], Yi Wang[2], Ping Chen[3], Qian Jiang[2], Ting Lan[4], Mei-Yu Li[3], Jin-Yu Su[3], Yan Wu[4], Jian Li[3]

[1]Department of Radiation Oncology, Affiliated Hospital of Jiangsu University, Zhenjiang, China. [2]Center of Experimental Medicine, Affiliated Hospital of Jiangsu University, Zhenjiang, China. [3]Department of Pulmonary Medicine, Affitialed Hospital of Jiangsu University, Zhenjiang, China. [4]Institute of Medical Science, Jiangsu University, Zhenjiang, Jiangsu, China.

Chun-Hau Dai and Yi Wang are co-first author.

Corresponding author:

Correspondence and requests for materials should be addressed to Jian Li. Department of Pulmonary Medicine, Affitialed Hospital of Jiangsu University, Zhenjiang, China.

E-mail: lijian541226@163.com

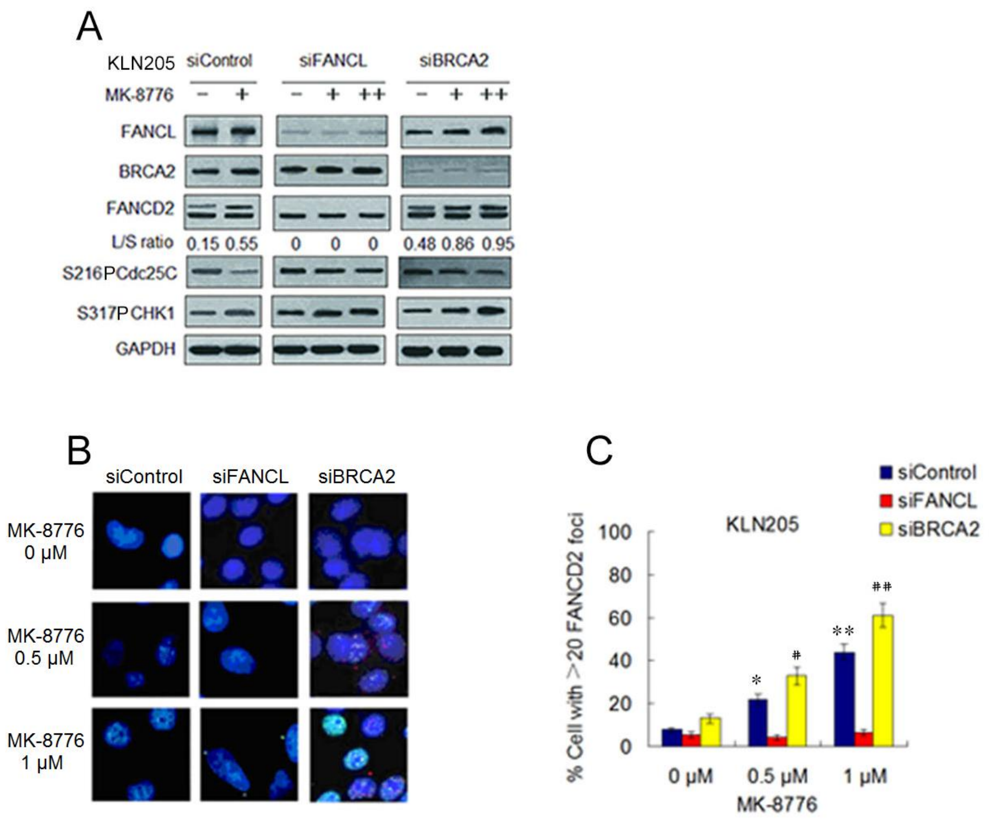

Supplementary Figure S1. (**A**) Western blots detecting monoubiquitination of FANCD2 and phosphorylation of Cdc25C (S216) in KLN205 cells transfected with siControl, siFANCD2 or siBRCA2. Cells were either treated with MK-8776 (0.5 µM +, 2 µM ++) or DMSO control for 6 h. GAPDH was used as loading control. (**B**,**C**) KLN205 cells were treated with MK-8776 at indicated doses following transfections with siFANCL or siBRCA2, fixed and immunostained using a anti-FANCD2 antibody. The percentage of cells with $>$ 20 FANCD2 foci was quantified using Spot Advanced RT Software (the cells treated with 0.5 µM MK-8776 as compared with the cells treated with 0 µM MK-8776, * $P < 0.05$, # $P < 0.01$; the cells treated with 1 µM MK-8776 as compared the cells treated with 0 µM MK-8776, ** $P < 0.01$, ## $P < 0.001$).

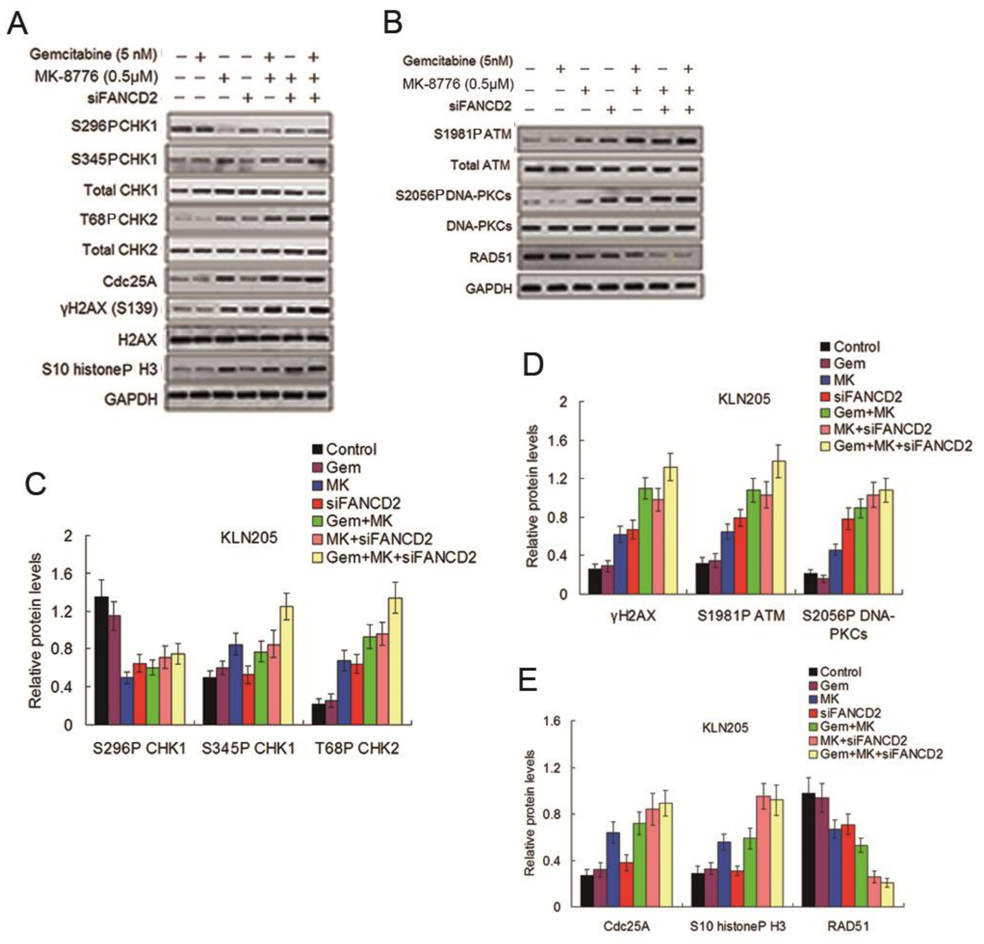

Supplementary Figure S2. (**A,B**) Before and after siFANCD2 transfection, the KLN205 cells were treated with gemcitabine for 4h, or MK-8776 for 6 h, or co-treated with gemcitabine and MK-8776 for 4 h and then with MK-8776 alone for another 6 h after removal of gemcitabine. Cell lysates were analyzed by Western blotting for the indicated proteins as described in the materials and methods, GAPDH was used as a loading control. GAPDH was used as loading control. (**C**) The intensity of S296P CHK1, S345P CHK1, T68P CHK2, and **(D)** γH2AX, S1981P ATM, and S2056P DNA-PKCs bands shown in **(A,B)** was quantified by densitometry and normalized against that of their nonphosphorylated bands. **(E)** The intensity of Cdc25A, S10 histoneP H3, and RAD51 bands was quantified by densitometry and normalized against that of the GAPDH bands.

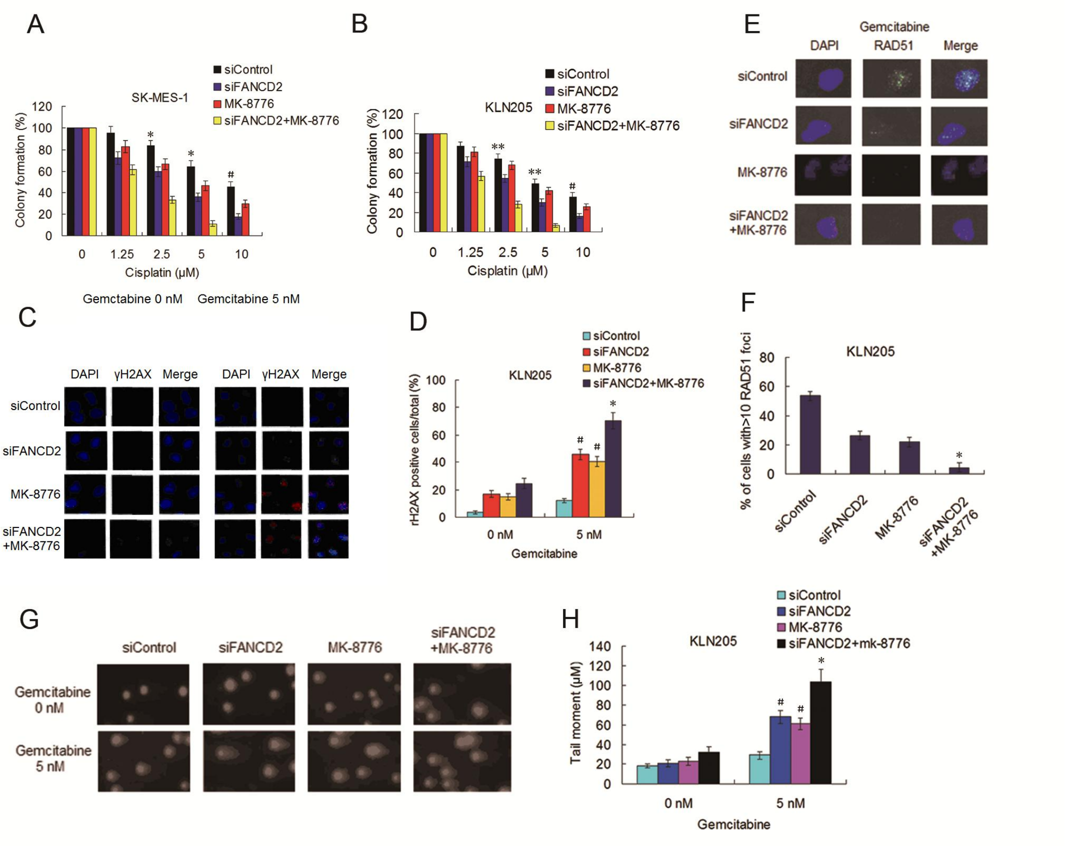

Supplementary Figure S3. (**A**) SK-MES-1 and (**B**) KLN205 cells were transfected with siFANCD2, or treated with MK-8776 for 6 h, or siFANCD2 transfection plus MK-8776. Then cells were exposured to cisplatin at various doses for 1 h and stained by crytal violet. Total colonies were counted after two weeks. Colony numbers of control were set as 100% (* siControl as compared with siFANCD2, $P < 0.05$, as compared with siFANCD2 plus MK-8776, * $P < 0.001$; ** siControl as compared with siFANCD2, $P < 0.01$, as compared with siFANCD2 plus MK-8776, $P < 0.001$; # siControl as compared with siFANCD2, $P < 0.005$, as compared with MK-8776, * $P < 0.05$;). (**C,D**) After transfection with siFANCD2, KLN205 cells were treated with gemcitabine, or MK-8776, or gemcitabine plus MK-8776, cultured in fresh medium for another 48 h, fixed and immuostained with an anti-γH2AX antibody. The percentage of γH2AX foci positive cells was quantified using Matafer Software (siFANCD2 plus MK-8776 as compared with siControl, * $P < 0.001$; compared with siFANCD2 or MK-8776 alone, * $P < 0.05$; siFANCD2 and MK-8776 as

compared with siControl, # $P < 0.01$). (**E,F**) After transfection with FANCD2, cells were treated with methods as described above, and fixed and immunostained with RAD51 antibody. The percentage of cells with >10 RAD51 foci was quantified from Image Software (siFANCD2 plus MK-8776 as compared with siControl, * $P < 0.001$; compared with siFANCD2 or MK-8776, * $P < 0.01$). (**G,H**) Cells were transfected with siFANCD2, and then treated with gemcitabine or MK-8776 alone, or with gemcitabine plus MK-8776. Alkaline comet assay was used to determine SSBs and DSBs, and the images show detectable comet tail when visualized under a fluorescent microscope. Tail moment in the cells were quantified using Comet Score Software version 1.5 (siFANCD2 plus MK-8776 as compared with siControl, * $P < 0.001$; compared with siFANCD2 or MK-8776 alone, * $P < 0.05$; siFANCD2 and MK-8776 as compared with siControl, # $P < 0.01$).

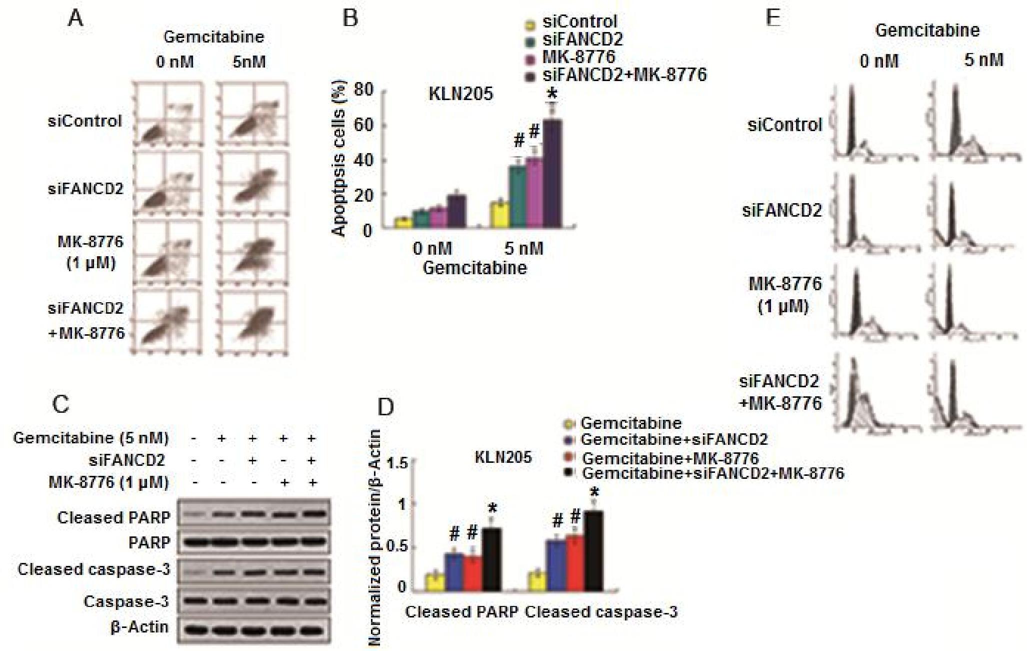

Supplementary Figure S4. (**A,B**) Cell apoptosis was detected by flow cytometry with Annexin γ-FITCIPI staining in KLN205 cells transfected with siFANCD2 before and after treatment with gemcitabine, or MK-8776, or gemcitabine plus MK-8776. Apoptosis rates were quantified by computer software (siFANCD2 plus MK-8776 as compared with siControl, * $P < 0.001$; compared with siFANCD2 or MK-8776, * $P < 0.05$; siFANCD2 or MK-8776 as compared with siControl, # $P < 0.01$). (**C,D**) Cleaved PARP and cleaved caspase-3 were determined by Western blotting in KLN205 cells before and after transfection with siFANCD2, or siFANCD2 plus gemcitabine, or treatment with gemcitabine or MK-8776 alone, or gemcitabine plus MK-8776 , or siFANCD2 combined with gemcitabine plus MK-8776. β-actin was used as loading control. (gemcitabine plus MK-8776 plus siFANCD2 as compared with gemcitabine alone, * $P < 0.005$; compared with gemcitabine plus siFANCD2 or gemcitabine plus MK-8776, * $P < 0.05$; gemcitabine plus siFANCD2 or gemcitabine plus MK-8776 as compared with gemcitabine alone, # $P < 0.05$). (**E**) Representative imagines of cell cycle analyses showed that MK-8776 reduced the S phase accumulations induced by gemcitabine, and co-treatment with MK-8776 and

siFANCD2 transfection increased sub-G1 population simultaneous with decrease of S phase accumulation in KLN205 cells.

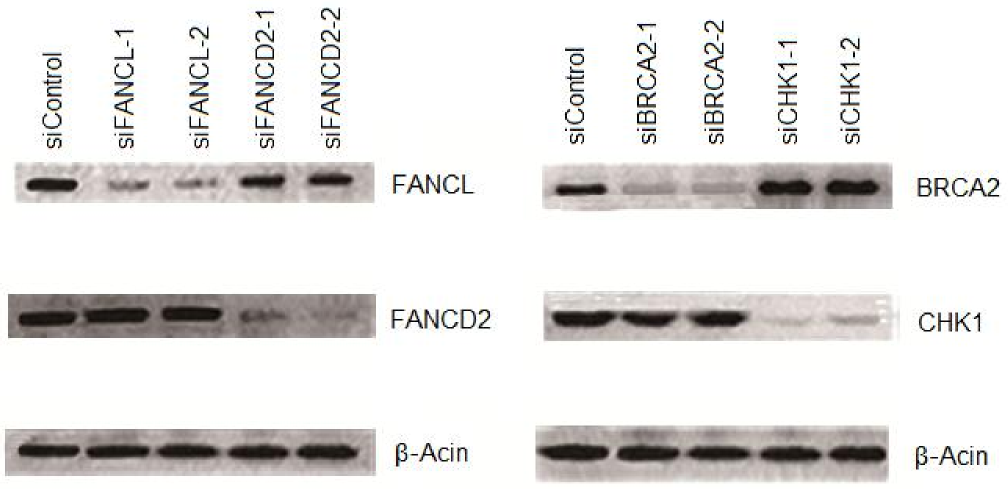

Supplementary Figure S5. Uncropped versions of the blots in Figure 1B.

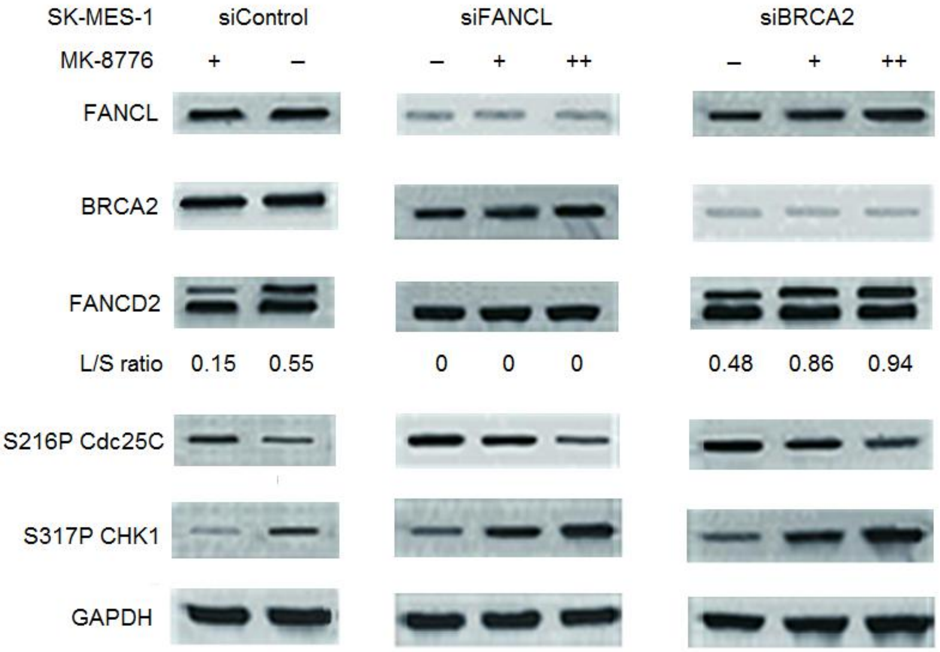

Supplementary Figure S6. Uncropped versions of the blots in Figure 2E.

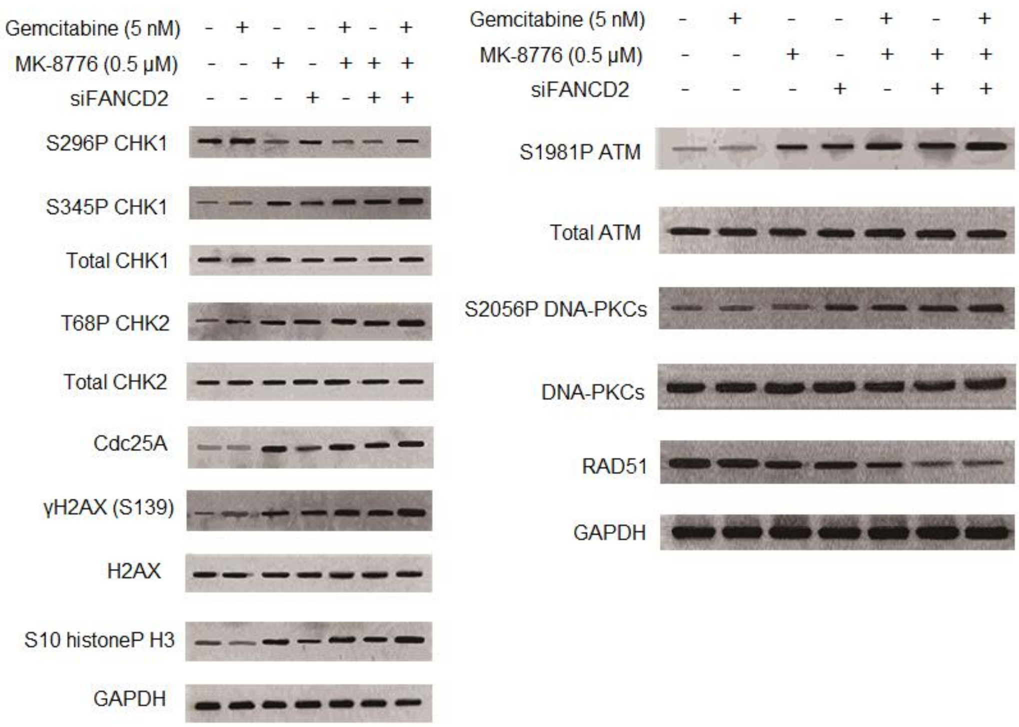

Supplementary Figure S7. Uncropped versions of the blots in (A) Figure 3A and (B) Figure 3B.

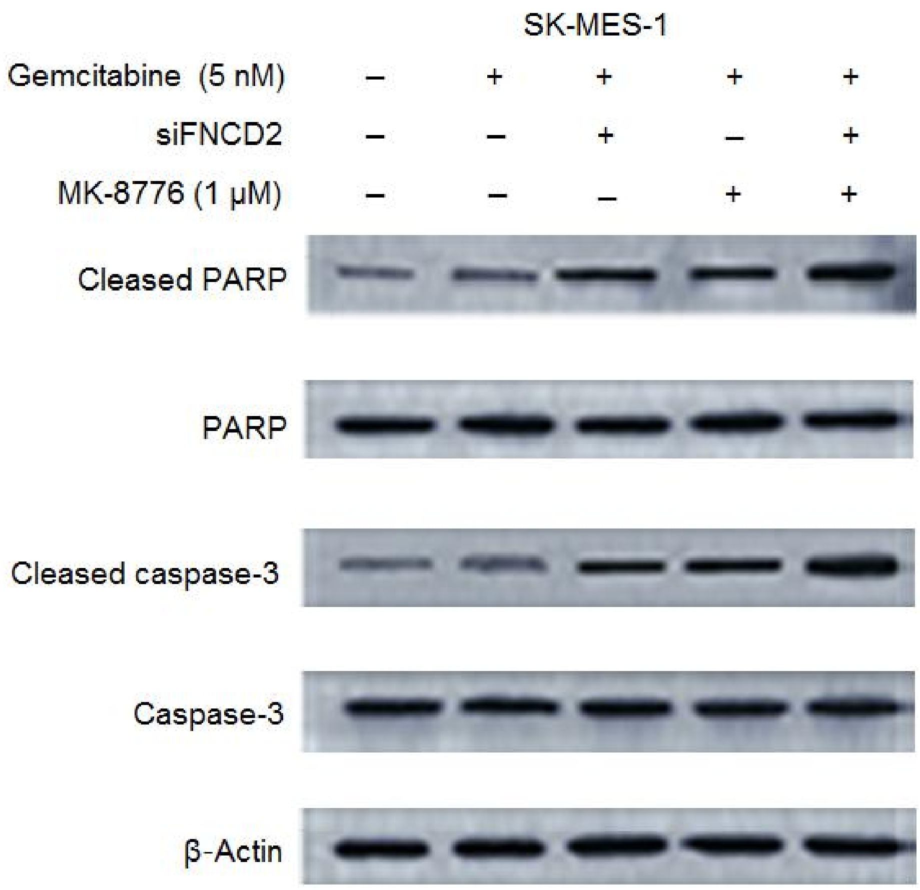

Supplementary Figure S8. Uncropped versions of the blots in Figure 6C.
